# Temporary Black Henna Tattoos and Sensitization to *para*-Phenylenediamine (PPD): Two Paediatric Case Reports and a Review of the Literature

**DOI:** 10.3390/ijerph14040421

**Published:** 2017-04-14

**Authors:** Elisa Panfili, Susanna Esposito, Giuseppe Di Cara

**Affiliations:** Pediatric Clinic, Università degli Studi di Perugia, 06129 Perugia, Italy; elisa.panfili@alice.it (E.P.); giuseppe.dicara@unipg.it (G.D.C.)

**Keywords:** allergic contact dermatitis, henna, *para*-phenylenediamine, temporary black henna tattoo

## Abstract

*Background*: The use of temporary henna tattoos has increased dramatically in recent years, especially in children and adolescents. To obtain a darker colour and prolong the life of the tattoo, red henna, a plant-derived substance, is typically added to *para*-phenylenediamine (PPD). The mixture is called temporary black henna tattoo (TBHT). Because of its molecular characteristics, PPD can induce skin sensitization that may cause various clinical manifestations with successive exposures, among which the most common is allergic contact dermatitis (ACD). This report describes two paediatric cases of PPD sensitization and ACD after the exposure to TBHT and summarizes the literature on this emerging clinical problem. *Case Presentation*: We describe two cases of childhood-onset ACD that occurred 2 and 10 days, respectively, after the application of a TBHT during the summer holidays. Patch tests showed an evident positive response to 1% PPD in both cases. Sensitization to PPD occurred in the first case because a previous henna tattoo did not result in overt symptoms; in the second case, the reaction occurred after the same tattoo was retouched. In both cases, hypopigmentation persisted and both the patients and their families were advised to avoid further contact with PPD-containing materials and substances that could lead to cross-reactions. *Conclusions*: Sensitization to PPD is a growing phenomenon in children. The most common cause appears to be exposure to TBHT in which PPD might be present at unknown or high concentrations. Once sensitization occurs, patients may experience severe clinical symptoms which can present with a persistent hypopigmentation when they are re-exposed to substances that contain or cross-react with PPD. Given the widespread use of PPD, TBHT could adversely affect the daily life of paediatric patients; thus, for this reason, this practice as a fashion accessory must be discouraged. In addition, it is extremely important to provide scientific information on the risks of TBHT to consumers, especially to adolescents and to the parents of younger children to prevent PPD sensitization.

## 1. Background

During the past 15 years, a new mode of henna application, the so-called temporary black henna tattoo (TBHT), has become fashionable among children, adolescents and young adults. Generally, these types of tattoos are applied to young people in holiday resort areas (e.g., in Southern Europe, Turkey, Egypt, United States, Mexico, Australia, Southeast Asia and South Africa), at theme parks, festivals and fairs by artisan street tattoo artists with mobile studios [1]. 

In nature, black henna does not exist, but is derived from red henna (an orange pigment extracted from the *Lawsonia inermis* plants with a low potential for sensitization) mixed with various substances [2], among which the most important is *para*-phenylenediamine (PPD; i.e., a dye, that when dispersed, results in an intense black shade) [3,4,5]. The addition of PPD is essential to make the henna paste darker, longer lasting, more beautiful and more similar to henna without PPD. When PPD is in its oxidized form and at low concentrations, it does not have sensitizing effects, however, PPD is often added in henna tattoos in a non-oxidized form and in unknown concentrations, which can induce skin sensitization. Currently, the European Union prohibits the use of PPD in any topical product, with the exception of hair dyes, which may contain up to 6% [6]. The most common clinical manifestation of PPD sensitization is allergic contact dermatitis (ACD), which presents with erythema, blisters and bubbles at the area of application of the substance [7,8,9]. In the cases of ACD from TBHT, the dermatitis takes peculiar forms that reflect the tattoo design that has been applied. Sometimes serious systemic allergic reactions may occur [10,11,12]. 

Although TBHTs are usually considered harmless and temporary, in some cases they can produce permanent consequences. In addition, PPD is present in many everyday substances, whose exposure should be avoided in patients who have been previously sensitized. TBHT-related sensitization was uncommon in the paediatric population up until a few years ago. However, due to the increased use of tattoos, this condition is becoming an emerging issue, even during the first years of life. This report describes two paediatric cases of PPD sensitization and ACD after exposure to TBHT and summarizes the literature on this emerging clinical problem. 

## 2. Case Presentation

A 9-year-old Caucasian male who was a native of Albania was evaluated at our paediatric clinic for the appearance of erythaematous and oedematous skin lesions with associated itching in the middle-distal area of the left arm, with well-defined margins that were eagle-shaped. The cutaneous clinical presentation had appeared two days after the application of TBHT, which was performed by a local tattoo artist during a summer holiday in Albania. 

**Figure 1:**
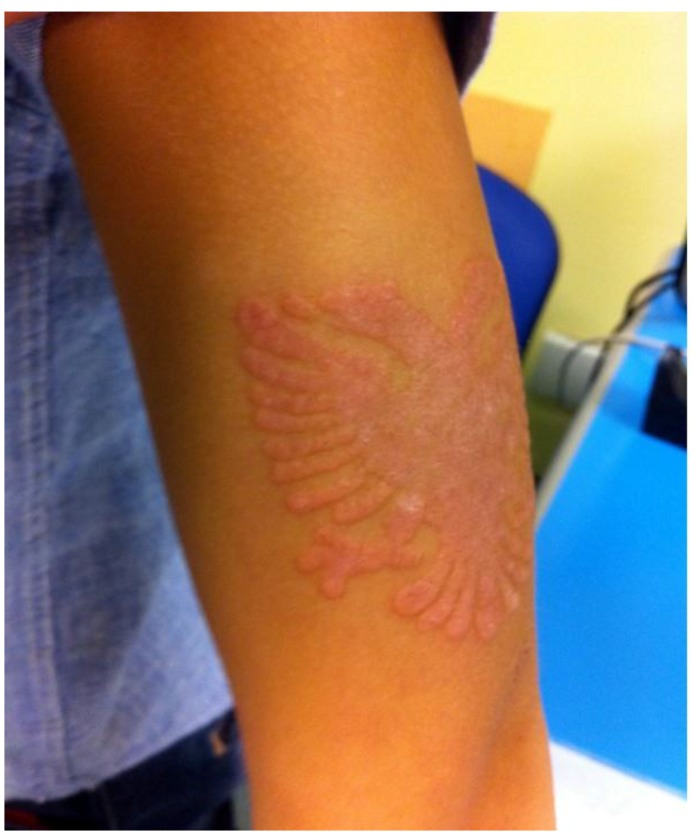
Case one: An eagle-shaped lesion in the middle-distal area of the left arm approximately 7 days after tattoo removal.

The patient’s personal history was negative for atopic dermatitis, defects in the skin barrier or allergies during the first years of life; the child had never previously presented with any similar skin reactions, despite having other henna tattoos applied previously (at least two times in previous summers). Given the onset of symptoms, the tattoo had been removed and therapy with a topical corticosteroid (betamethasone dipropionate 0.05%) had been initiated based on the advice of a local doctor; the treatment led to partial improvement in the itching. A week after beginning the corticosteroid therapy, the skin lesions were still present (Figure 1). 

Considering the time that had elapsed between the application of the tattoo, the clinical manifestations and the possible previous sensitization to PPD, a clinical suspicion of ACD secondary to TBHT in a patient sensitized to PPD was suggested, and patch tests were scheduled. Meanwhile, the patient started therapy with an oral antihistamine (levocetirizine) and topical corticosteroids (budesonide 0.025%). After two weeks, the skin lesions appeared to be improved, although they had not totally disappeared. Four weeks after the end of the topical therapy, the patient underwent clinical follow-up: when the skin was evaluated, a discoloured lesion persisted at the tattoo site.

Patch testing for chemical substances was conducted using the AtopyLine products (Euromedical, Brescia, Italy) with re-evaluation of the patient at 48 h and 72 h after application. After 48 h, the reaction to PPD 1% showed a vesicular lesion (++), whereas after 72 h, an ulcerative lesion (+++) was present, which confirmed the clinical suspicion of a PPD sensitization. After one year of follow-up, the patient was in good general clinical condition with an area of hypopigmentation of the skin that was still evident from when the patient had presented with ACD from the TBHT. 

**Figure 2:**
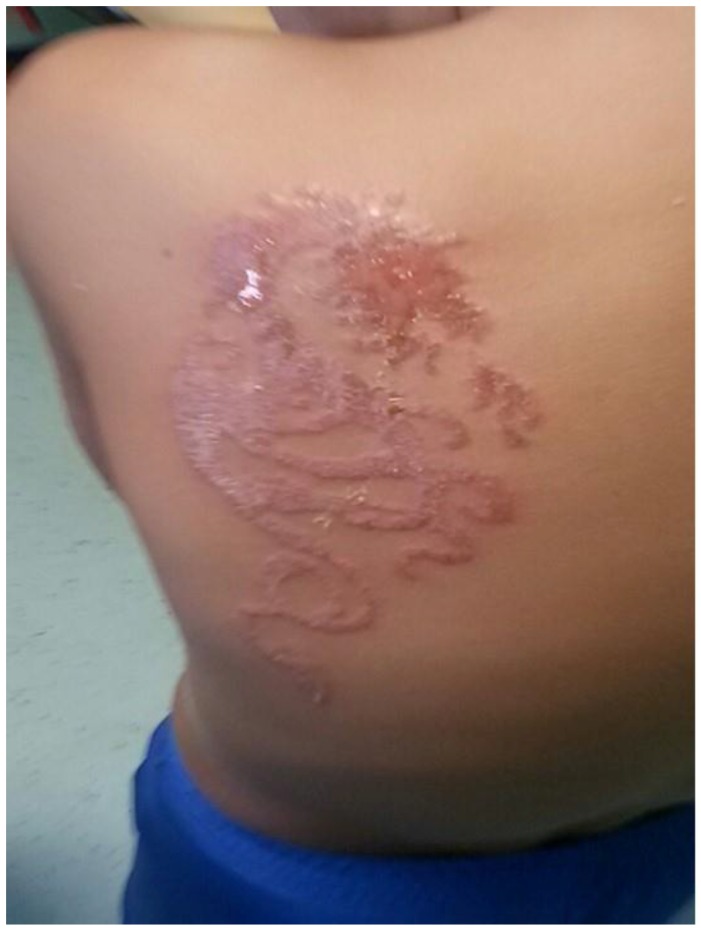
Case two: A large skin lesion depicting a dragon on the left lower back. The patient presented with an erythaematous skin lesion in the peripheral zone and papular-vesicular and exudative lesions in the central areas.

In the second case an 8-year-old Italian male who was evaluated at our centre for the appearance of a large erythaematous and extremely itchy skin lesion on the left, lower back that depicted a dragon, with papular-vesicular lesions and exudative elements in the central area that had well-defined margins (Figure 2). 

The child had returned from a vacation to Egypt with his family; approximately 10 days before returning home, he had received a TBHT depicting a dragon by a local tattoo artist on the beach. At 48 h after the first application of the tattoo, the patient did not present with any symptoms, but shortly before returning to Italy he had undergone retouching of the colour tattoo. The personal and family history was negative for allergic skin diseases or disorders; the patient had never received henna tattoos before, and he had never been in contact with materials that could contain PPD, although the mother was a hairdresser. 

A topical cortisone therapy was administered for 3 weeks and an oral antihistamine was used for 2 weeks. With this treatment, itching resolved and the skin lesions were significantly reduced. 

One month after the discontinuation of topical steroid therapy, a patch test for chemical substances (Euromedical) was performed and showed a positive reaction to PPD 1%, with the appearance of erythaematous vesicular-ulcerative lesions (+++) that appeared within 72 h; the other substances were negative. A diagnosis of ACD from TBHT in a patient with de novo sensitization to PPD was delivered. After one year, the patient, who was in a good clinical condition, still presented with a hypopigmentation of the skin and meticulously avoided contact with substances that could contain PPD.

These case reports were approved by the Ethics Committee of Azienda Ospedaliera di Perugia, Perugia, Italy. For the case reports, Azienda Ospedaliera di Perugia, Perugia, Italy, does not provide a reference number.

## 3. Discussion 

These two paediatric cases showed an ACD and a PPD sensitization following TBHT, which is atypical in paediatric patients <14 years. In the first case, the temporal distance between the application and the onset of symptoms was short (2 days) due to the probability of a previous asymptomatic exposure to PPD. In the second case, the reaction time was more delayed (10 days after the first application) and was triggered by a retouch of the same tattoo. Both patch tests were positive for PPD 1%, which confirmed the sensitization of the patients to this substance. However, in the second case, the profession of the mother was highly suspicious for potential previous accidental, environmental exposure to PPD and represented a potential risk for further possible contact and worsening of the patient’s symptoms. 

Compared to a permanent tattoo, TBHT is commonly considered to be more advantageous because there is no associated pain or risk of infection, and although it leaves a mark, it disappears after a few weeks and can be reapplied if desired [13,14,15]. For all these reasons, parents who authorize their children to receive the tattoos consider it a natural practice. Indeed, because of this easy usage, this practice is often conducted by unregulated tattoo artists. Moreover, because the mixture of products is generally prepared extemporaneously by the artist, the variety of materials and the concentrations of PPD and other ingredients are unknown. Skin reactivity following an exposure to PPD that results from TBHT is a phenomenon of growing importance in the paediatric population. Once sensitized, paediatric patients can experience ACD or severe clinical manifestations later in life from contact with other PPD-containing products that include black hair dye, leather shoes, fur, textiles, nylon, rubber, paints, photographic prints and industrial printing inks. This wide range of potential exposure makes it difficult for sensitized patients to completely avoid further contact in real-life and limits the choice of profession in sensitized patients. High-risk jobs include being a hairdresser, a furrier, a shoe salesman, or a chemical worker and includes working in printing companies, the leather industry, and rubber and textile production. As we completed with our cases, it is extremely important to inform the patients and their families of products that should be avoided. 

Several studies have investigated the presence and the amount of PPD in black henna preparations and have showed that the data varies greatly among countries [16,17,18,19]. In the United States, the Food and Drug Administration has found PPD concentrations in henna ranging from 4.9% to 27.2% [19]. In addition to the high and, in some cases, unknown concentrations of PPD in henna tattoos, there are other elements that can promote sensitization: prolonged contact with the skin and the lack of an oxidizing substance that neutralizes the effect; the use of oils or solvents; a hot environment that increases the local temperature of the skin facilitating the percutaneous penetration; the use of syringes to apply the dye; the use of occlusive dressings to keep the henna attached to the skin; and the retouching of tattoos [15,20]. Some of these factors could have played a role in both of our cases.

Most of the patients who exhibit an allergic reaction become sensitized to PPD during the application of the tattoo itself. The time between the application of the tattoo and the onset of symptoms is variable and usually ranges from 4 to 7 days in cases of de novo sensitization [20,21]. Our first case developed a reaction before the accepted range, which could be explained by the likelihood of a previous sensitization; whereas, in the second case, the retouching of the tattoo stimulated the sensitization. Nevertheless, in patients who have reactions after an exposure to TBHT, it is extremely important to analyse the previous exposure to products that may have contained PPD [22,23,24].

From a clinical perspective, our cases presented with typical ACD, which is characterized by erythema, oedema, papules and vesicles and is relatively aggressive albeit limited to the site of the tattoo with well-defined margins, which is comparable to tattooing [25]. Other possible manifestations are lichenoid dermatitis, erythema multiforme, urticaria, angio-oedema or systemic symptoms such as lymphadenopathy or fever [26,27,28,29,30,31,32,33]. However, long-term events include post-inflammatory hypopigmentation, which also occurred in our two patients and in some cases, may be permanent [34]. 

The gold standard for the diagnosis of ACD to PPD is the patch test [35,36]. After a suspected diagnosis based on clinical history and cutaneous manifestations, all patients should be evaluated using a patch test. In the case of children and adolescents with a history of allergic reactions to henna tattoos, some authors recommend starting with ≤0.05% PPD concentration, which is titrated to a maximum of 1% [14]. The patch test should be evaluated after 48–72 h, and in some cases, even after 96 h. The reactions should be interpreted as negative (no signs), 1+ (erythema, papules), 2+ (vesicles), and 3+ (ulcerations) [14]. Other authors argue that in the case of a strong clinical suspicion it is not necessary to perform the patch test for PPD [37]. However, when the patch test is not performed, there may be lifetime consequences regarding the patient’s choice of profession and the possibility of avoiding certain substances. 

In a Spanish study, the authors highlighted that sensitization to PPD in the past 15 years has increased in the paediatric population secondary to the introduction of TBHT [38]. The study was conducted between 1980 and 2015 by the General Allergology Department at the University Hospital of Valencia. Among 726 children who were 0 to 16 years old, 34 cases (4.7%, mean age 12.4 years) were sensitized to PPD. Of these 34 patients, 25 (73.5%) had major reactions with involvement of the arms (32% of cases) and abdomen (13% of cases). The patient’s history showed that sensitization had occurred in 50% of cases (17 patients) with the application of a henna tattoo, in 11.7% (4 patients) with use of a hair dye, in 2.9% (1 patient) with the application of lipstick and in 35.2% (12 patients), the cause remained unknown. These authors also showed that the numbers of PPD allergies have increased significantly over time: 22 cases in the period from 2000 to 2015 compared with 9 in the period between 1985 and 2000 [38].

In the United States, the law does not permit the import of temporary tattoos containing PPD; therefore, the use of black henna is considered illegal; similar directives have also been applied in other countries, such as Canada, New Zealand and Australia [39]. However, in European countries, there are no definitive statements that regulate the practice of henna tattoo application, making difficult to monitor the distribution of these products and to regulate tattoo artists who practice on beaches and in markets, fairs and amusement parks.

## 4. Conclusions 

Our cases show that TBHT-related adverse events are emerging problems in the paediatric patient population. Given the widespread use of PPD, TBHT could adversely affect the daily life of paediatric patients; thus, for this reason, the use of TBHT for fashion must be discouraged because it is potentially dangerous to the health of children and heavily conditions their future personal career choices. In addition, it is extremely important to provide scientific information on the risks of TBHT to consumers, especially to adolescents and to the parents of younger children to prevent PPD sensitization.
